# Metformin inhibits spontaneous excitatory postsynaptic currents in spinal dorsal cord neurons from paclitaxel-treated rats

**DOI:** 10.3389/fnsyn.2023.1191383

**Published:** 2023-05-05

**Authors:** Ting-Ting Liu, Chun-Yu Qiu, Wang-Ping Hu

**Affiliations:** ^1^School of Basic Medical Sciences, Xianning Medical College, Hubei University of Science and Technology, Xianning, Hubei, China; ^2^Department of Physiology, Hubei College of Chinese Medicine, Jingzhou, Hubei, China

**Keywords:** metformin, paclitaxel, sEPSCs, nociception, CIPN

## Abstract

**Introduction:**

Cancer patients treated with paclitaxel often develop chemotherapy-induced peripheral neuropathy, which has not been effectively treated with drugs. The anti-diabetic drug metformin is effective in the treatment of neuropathic pain. The aim of this study was to elucidate effect of metformin on paclitaxel-induced neuropathic pain and spinal synaptic transmission.

**Methods:**

Electrophysiological experiments on rat spinal slices were performed *in vitro* and mechanical allodynia quantified *in vitro*.

**Results:**

The present data demonstrated that intraperitoneal injection of paclitaxel produced mechanical allodynia and potentiated spinal synaptic transmission. Intrathecal injection of metformin significantly reversed the established mechanical allodynia induced by paclitaxel in rats. Either spinal or systemic administration of metformin significantly inhibited the increased frequency of spontaneous excitatory postsynaptic currents (sEPSCs) in spinal dorsal horn neurons from paclitaxel-treated rats. We found that 1 h incubation of metformin also reduced the frequency rather than the amplitude of sEPSCs in the spinal slices from paclitaxel-treated rats.

**Discussion:**

These results suggested that metformin was able to depress the potentiated spinal synaptic transmission, which may contribute to alleviating the paclitaxel-induced neuropathic pain.

## 1. Introduction

Paclitaxel is a frontline clinical drug used to treat many solid tumors (Hagiwara and Sunada, 2004). A common dose-limiting adverse side effect of paclitaxel is chemotherapy-induced peripheral neuropathy (CIPN), which can result in severe acute and chronic pain during paclitaxel treatment and after withdrawal (Han and Smith, 2013; Sisignano et al., 2014). So far, there is still a lack of effective drugs to treat the paclitaxel-induced neuropathic pain in clinic (Burgess et al., 2021). Spinal central sensitization is an important mechanism underly neuropathic pain, which is characterized by enhanced synaptic transmission in the spinal dorsal horn neurons (Woolf, 2011). There is evidence that excitatory synaptic transmission in spinal dorsal horn neurons is enhanced in CIPN, which involves increased excitability of primary sensory afferent neurons (Li et al., 2015). Electrophysiological recordings show that the frequency of excitatory postsynaptic currents (EPSCs) of dorsal horn neurons is increased in paclitaxel-treated rats (Xie et al., 2016).

Metformin is a drug widely used to treat type II diabetes. Metformin has been found to reverse the established mechanical hyperalgesia in a variety of neuropathic pain, such as pain caused by spinal cord nerve ligation and spared nerve injury (Melemedjian et al., 2011, 2013). In addition, metformin can also prevent neuropathic pain caused by chemotherapy (Mao-Ying et al., 2014; Inyang et al., 2019). It is still unclear whether metformin alleviates paclitaxel-induced neuropathic pain by affecting synaptic transmission in the spinal dorsal horn neurons. The present study indicated that spinal dorsal horn neurons displayed enhanced spontaneous excitatory postsynaptic currents (sEPSCs) in paclitaxel-treated rats, which was significantly inhibited by intrathecal and systemic administration of metformin in rats and incubation of metformin in spinal slices. Intrathecal administration of metformin also reversed the paclitaxel-induced mechanical allodynia.

## 2. Materials and methods

### 2.1. Animals

Male Sprague–Dawley rats (3–4 weeks of age) were housed in individual cages. The rats had free access to food and water. Room temperature was controlled at 23 ± 2°C during a 12 h light/dark cycles. All animal experimental protocols were approved by the Animal Research Ethics Committee of Hubei University of Science and Technology (2016-03-005). To evaluate the effect of intrathecal treatment with metformin on sEPSCs in spinal dorsal horn neurons from paclitaxel-treated rats, animals were randomly divided into three groups (*n* = 18, 6 rats in each group). In control group (CON), rats were injected corresponding vehicle intraperitoneally and intrathecally, respectively. In paclitaxel-treated group (PAC), rats received intraperitoneal injection (i.p.) of paclitaxel (2 mg/kg per injection at day 1, 3, 5, and 7) and intrathecal injections (i.t.) of 50 μl vehicle. In paclitaxel-metformin (i.t.)-treated group (MET), metformin (100 ug in 50 ul, at day 8, 9, and 10) was intrathecally treated in paclitaxel-treated rats. To further investigate the effect of systemic treatment with metformin on sEPSCs in spinal dorsal horn neurons from paclitaxel-treated rats, rats were randomly divided into three groups (*n* = 18, 6 rats in each group). In control group (CON), rats were injected corresponding vehicle intraperitoneally. In paclitaxel-treated group (PAC), rats received intraperitoneal injection (i.p.) of paclitaxel (2 mg/kg per injection at day 1, 3, 5, and 7) and 50 μl vehicle (i.p. at day 8, 9, and 10). In paclitaxel-metformin (i.p.)-treated group (MET), metformin (200 mg/kg, at day 8, 9, and 10) was intraperitoneally treated in paclitaxel-treated rats. At day 11 or 12, spinal cord slices were prepared to record sEPSCs in all rats. To study the acute effect of metformin on sEPSCs, spinal slices (at day 11 or 12) from paclitaxel-treated rats were incubated by oxygenated ACSF containing metformin (2 mM) or vehicle for 1 h, and then sEPSCs were recorded.

### 2.2. Intrathecal Surgeries and drug application

Intrathecal catheters were implanted on rats 15 days before paclitaxel treatment. Under deep anesthesia with a mixture of ketamine (75 mg/kg) and medetomidine (0.5 mg/kg), rats were inserted a polyethylene-10 catheter into their subarachnoid space through L5–L6 intervertebral space, and the tip of the catheter was located at the L5 spinal segmental level. To establish a mechanical allodynia model, rats received intraperitoneal injection (i.p.) of paclitaxel (Sigma-Aldrich, 2 mg/kg) at day 1, 3, 5, and 7, with a cumulative dose of 8 mg/kg. Paclitaxel was dissolved in a 1/1 Cremophor EL (Sigma-Aldrich)/ethanol solution then further prepared with 0.9% saline for injection. Rats in the control group received the vehicle (Cremophor EL/ethanol, 1:1) on the same four alternate days. Metformin (Sigma-Aldrich) was treated at day 8, 9, and 10. Metformin was dissolved in artificial cerebrospinal fluid and delivered through the intrathecal catheter. Metformin was also dissolved in 0.9% saline and intraperitoneally injected. After paclitaxel and metformin were applied, rats were sacrificed to prepare spinal cord slices for electrophysiological recordings at day 11 or 12.

### 2.3. Spinal slice preparations

The rats were anesthetized with 1.5 mg/kg urethane (10%, i.p.). A laminectomy was performed, and the lumbar segment was quickly removed and immersed in oxygenated (95% O_2_ and 5% CO_2_) cold ACSF. The ACSF contained 95.0 mM NaCl, 1.8 mM KCl, 0.5 mM CaCl_2_, 7.0 mM MgSO_4_, 1.2 mM KH_2_PO_4_, 26.0 mM NaHCO_3_, 15.0 mM Glucose and 50.0 mM Sucrose. Its pH was adjusted to 7.3–7.4 with NaOH and osmolarity to 310–320 mOsm/L with sucrose. The pial-arachnoid membrane was removed from the section. The L5 spinal segment was fixed on the cutting bracket with cyanoacrylate glue, then, 350 μm thick spinal cord slices were cut with a vibratome (Leica VT1200S Microsystem). Slices were incubated in ACSF oxygenated at 35°C for at least 1 h and then transferred to the recording chamber.

### 2.4. Electrophysiological recordings

The slice was placed into a recording chamber and continuously perfused with recording solution at a speed of 3–4 ml/min. The recording solution contained 127.0 mM NaCl, 1.8 mM KCl, 2.4 mM CaCl_2_, 1.3 mM MgSO_4_, 1.2 mM KH_2_PO_4_, 15.0 mM glucose, and 26.0 mM NaHCO_3_, oxygenated with 95% O_2_, and 5% CO_2_, at a pH of 7.3–7.4, and an osmolarity of 300–310 mOsm/L. Recordings of sEPSCs were performed using whole-cell voltage-clamp. All recordings were conducted in lamina II neurons at the L5 spinal level. Lamina II outer zone was identified by its distinctive translucent appearance and neurons were visualized using a water-immersion objective on an upright infrared Olympus microscope (BX51WI, Japan) with differential interference contrast/infrared illumination. sEPSCs were recorded using an EPC-10 amplifier and PULSE program (HEKA Electronics, Lambrecht, Germany). The recording pipette had a resistance of 8–10 MΩ when filled with the internal solution containing: 140.0 mM K-gluconate, 3.0 mM KCl, 4.0 mM NaCl, 0.2 mM EGTA, 10 mM HEPE and 2.0 mM Mg-ATP. Only a seal resistance of ≥ 2 GΩ and an access resistance of 20–35 MΩ were used for electrophysiological recording. The series resistance was optimally compensated by ≥70% and constantly monitored throughout the experiments. In the presence of 10 μM bicuculline and 5 μM strychnine, sEPSCs were recorded from neurons clamped at −70 mV. After a 15 min stabilization period, synaptic events were recorded for 4 min in every slice. A spinal cord slice was obtained from each animal and only a neuron was recorded in a slice.

### 2.5. Behavioral tests

Male rats were placed in a Plexiglas chamber and adapted to the environment for at least 30 min before behavior experiments. Mechanical allodynia was measured by paw withdrawal threshold (PWT). After rats were coded and pretreated with paclitaxel, different groups of rats then received intrathecal injection of vehicle or different dose (10, 30, and 100 ug in 50 ul, i.t. at day 8, 9, and 10) of metformin, separately. After 30 min metformin injection, the other experimenter tested PWT of the hind plantar using a series of von Frey filaments (Stoelting, Wood Dale, IL, USA). Experimenters who performed behavioral tests were blinded to all treatments.

### 2.6. Data analysis

Spontaneous events were performed and analyzed using Mini Analysis Program version 6.0.3 (Synaptosoft, Decatur, GA, USA). Analysis was performed in Igor pro6.10A software. Data were expressed as mean ± SEM and statistically compared using unpaired *t*-test or analysis of variance (ANOVA), followed by Bonferroni’s *post-hoc* test.

## 3. Result

### 3.1. Paclitaxel-induced mechanical allodynia is prevented by intrathecal injection of metformin

**FIGURE 1:**
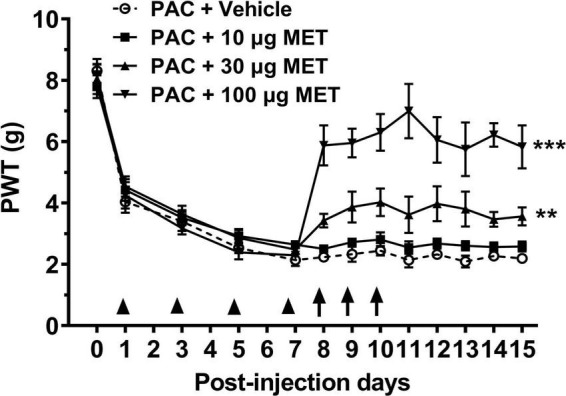
Intrathecal injection of metformin reverses paclitaxel-induced mechanical allodynia in rats. Treatment with paclitaxel (8 mg/kg at day 1, 3, 5, and 7) significantly reduced the paw withdraw threshold (PWT, in grams) in rats. Intrathecal injection of metformin (at day 8, 9, and 10) prevented paclitaxel-induced mechanical allodynia in a dose-dependent manner. *N* = 10 rats in each group, **P < 0.01, ***P < 0.001, two-way ANOVA followed by Bonferroni *post-hoc* test, compared with PAC + Vehicle group.

To evaluate the effect of metformin on paclitaxel-induced mechanical allodynia, rats were intrathecally injected with different dose of metformin versus vehicle, and then their mechanical thresholds were compared. We observed that rats displayed mechanical allodynia after administration with paclitaxel (4 × 2 mg/kg, cumulative dose 8 mg/kg). Figure 1 shows that the mechanical withdrawal thresholds of rats were decreased 1 day after paclitaxel injection. The decrease was further intensified, which was significantly different from baseline value at day 0. Since day 7, withdrawal threshold of paclitaxel-treated rats remained stable at low levels throughout the experiment. Figure 1 shows intrathecal administration of metformin (10, 30, and 100 ug in 50 ul, i.t. at day 8, 9, and 10) dose-dependently prevented the paclitaxel-induced mechanical allodynia (*P* < 0.01 and 0.001, Bonferroni’s *post-hoc* test, compared with vehicle, *n* = 10 rats). Mechanical allodynia only partially developed in the paclitaxel and 100 ug metformin-treated rats, which was significantly less than that of the paclitaxel–vehicle-treated rats at days 8–15. These results suggested that intrathecal injection of metformin dose-dependently prevents the established mechanical allodynia induced by paclitaxel in rats.

### 3.2. Intrathecal treatment with metformin inhibits the increased frequency of sEPSCs in spinal dorsal horn neurons from paclitaxel-treated rats

**FIGURE 2:**
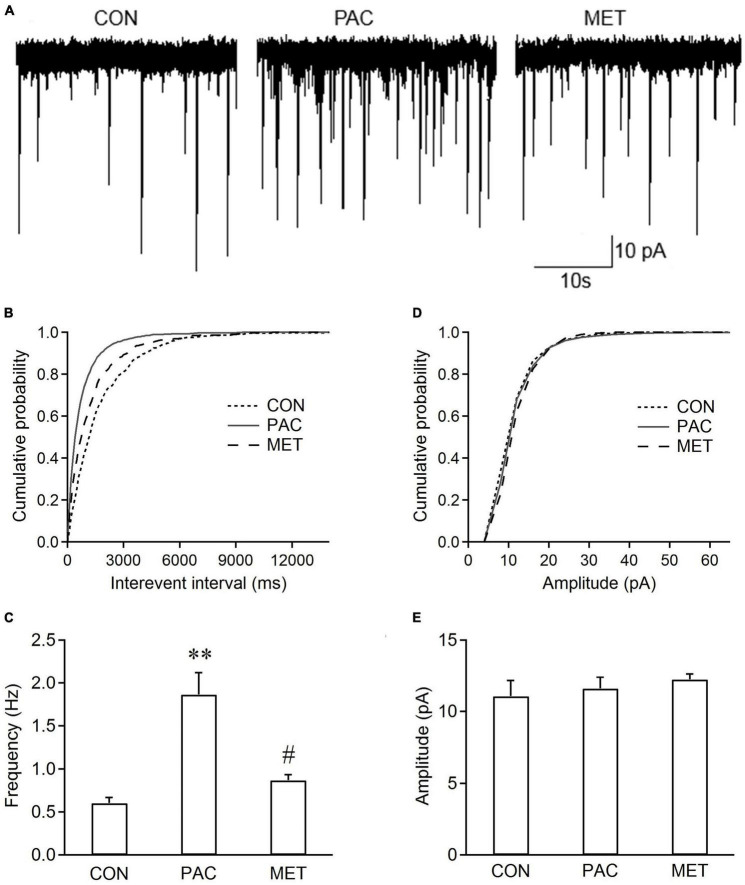
Intrathecal injection of metformin decreases the frequency rather than the amplitude of sEPSCs in spinal cord slices from paclitaxel-treated rats. **(A)** Typical traces of sEPSC recordings in spinal cord slices from control (CON), paclitaxel-treated (PAC), and paclitaxel-metformin (i.t.)-treated (MET) rats. Metformin (Met, 100 ug in 50 ul) was intrathecally injected daily in paclitaxel-treated rats at day 8, 9, and 10. Cumulative distributions **(B,D)** and the bar graphs **(C,E)** show the frequency rather than the amplitude of sEPSCs increased in spinal dorsal horn neurons from paclitaxel-treated rats, The increased sEPSC frequency recorded in slices from paclitaxel-treated rats was significantly decreased by intrathecal injection of metformin. However, metformin had no effect on sEPSC amplitude. Data are expressed as means ± S.E.M. *N* = 6 neurons from 6 rats, one-way ANOVA followed by Bonferroni *post-hoc* test, **P < 0.01, compared with control rats, ^#^*P* < 0.05, compared with paclitaxel-treated rats. V_H_ = –70 mV.

At day 11 or 12, sEPSCs in spinal dorsal horn neurons were recorded. Figure 2A shows the sEPSCs traces from three groups of rats. Cumulative distributions shows that the curve of sEPSC interevent interval was significantly shifted leftward in paclitaxel-treated (PAC) rats (Figure 2B), but no change in the curve of sEPSC amplitude (Figure 2D), compared with those from control (CON) rats. Figures 2C, E shows that sEPSC frequency was significantly increased (1.86 ± 0.26 Hz vs. 0.6 ± 0.07 Hz, *P* < 0.01, *n* = 6 neurons from 6 rats) without change in amplitude with paclitaxel, compared with those from control treatment. Metformin reversed this effect (Figures 2B, C). Intrathecal treatment with metformin significantly inhibited the increased frequency of sEPSCs with paclitaxel, as observed in paclitaxel-metformin treated (MET) rats (0.87 ± 0.07 Hz vs. 1.86 ± 0.26 Hz, *P* < 0.05, *n* = 6 neurons from 6 rats; Figures 2B, C), but not affected the sEPSC amplitude (Figures 2D, E). The results indicated that intrathecal administration of metformin inhibited the increased frequency of sEPSCs in spinal dorsal horn neurons from paclitaxel-treated rats.

### 3.3. Incubation of metformin reduces sEPSC frequency in spinal dorsal horn neurons from paclitaxel-treated rats

**FIGURE 3:**
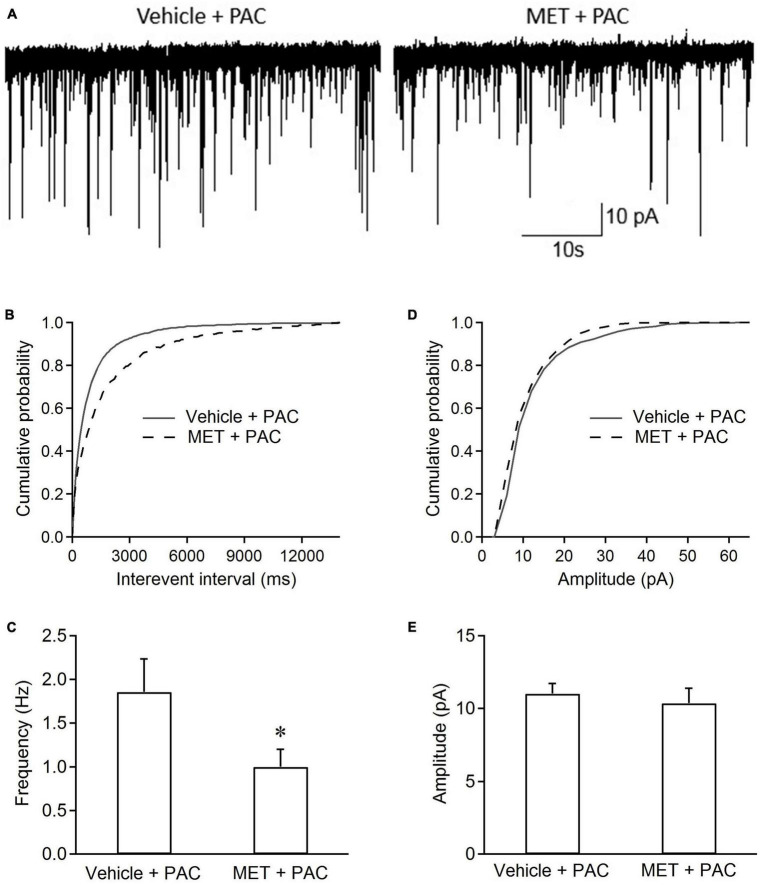
Incubation of metformin inhibits the frequency of sEPSCs in spinal dorsal horn neurons from paclitaxel-injected rats. **(A)** Typical recording traces of sEPSCs in spinal cord slices incubated with ACSF containing vehicle or metformin. Cumulative distributions **(B,D)** and the bar graphs **(C,E)** show incubation ACSF containing metformin (2 mM) decreased the frequency rather than the amplitude of EPSCs in spinal dorsal horn neurons from paclitaxel-treated rats. Data are expressed as means ± S.E.M. *N* = 6 neurons from 6 rats, *P < 0.05, unpaired *t*-test, compared with vehicle + PAC. V_H_ = –70 mV. All slices (at day 10 or 11) from paclitaxel-treated rats were pre-incubated in ACSF containing metformin or vehicle for 1 h at 35°C.

Figure 3 shows that incubation of slices with metformin for 1 h had also effects on sEPSCs in spinal dorsal horn neurons from paclitaxel-treated rats. Cumulative distributions shows that metformin incubation significantly shifted the curve of sEPSC interevent interval rightward and had no effects on the curve of sEPSC amplitude, indicating that metformin made the intervals between sEPSC events longer, but not significantly changed sEPSC amplitude, compared with vehicle incubation (Figures 3B, D). Figures 3B, D show that metformin incubation also significantly reduced the sEPSC frequency (0.99 ± 0.19 Hz vs. 1.75 ± 0.38 Hz, *P* < 0.05, *n* = 6 neurons from 6 rats), rather than sEPSC amplitude, in the slices from paclitaxel-treated rats. These results indicated that metformin had an acute effect on sEPSCs in spinal dorsal horn neurons from paclitaxel-treated rats.

### 3.4. Systemic treatment with metformin reduces the increased frequency of sEPSCs in spinal dorsal horn neurons from paclitaxel-treated rats

**FIGURE 4:**
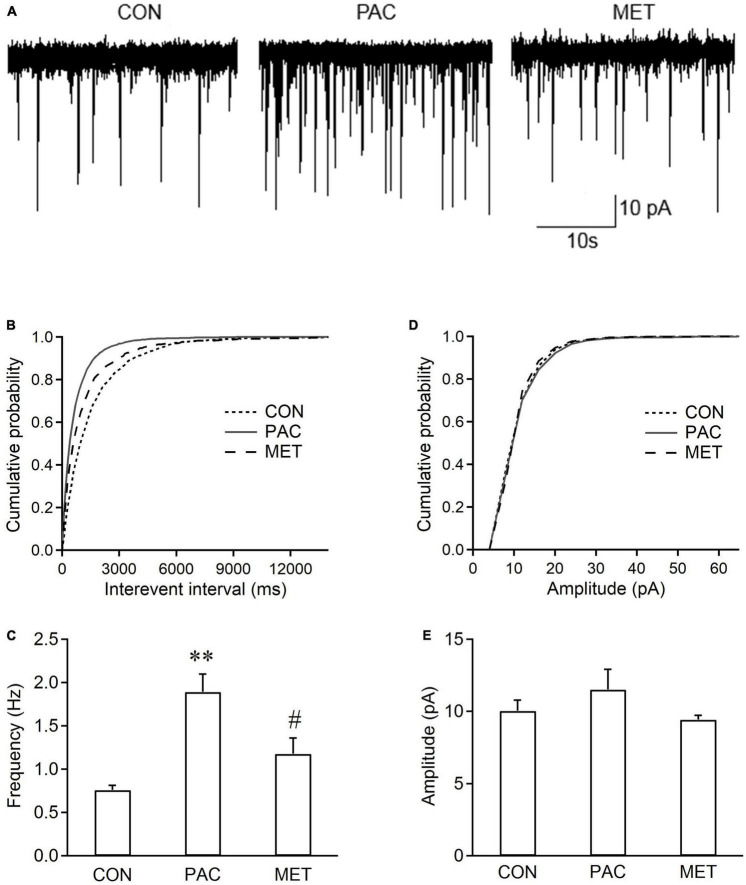
Systemic metformin inhibits the frequency of sEPSCs in spinal dorsal horn neurons from paclitaxel-injected rats. **(A)** Typical recording traces of sEPSCs in spinal dorsal horn neurons from control (CON), paclitaxel-treated (PAC, i.p.), and paclitaxel (i.p.)-metformin (i.p.)-treated (MET) rats. Metformin (Met, 200 mg/kg) was intraperitoneally injected daily in paclitaxel-treated rats at day 8, 9, and 10. Cumulative distributions **(B,D)** and the bar graphs **(C,E)** show systemic administration of metformin reduced the frequency rather than the amplitude of sEPSCs in spinal dorsal horn neurons from paclitaxel-treated rats. Data are expressed as means ± S.E.M. *N* = 6 neurons from 6 rats, one-way ANOVA followed by Bonferroni *post-hoc* test, **P < 0.01, compared with control rats, ^#^*P* < 0.05, compared with paclitaxel-treated rats. V_H_ = –70 mV.

Figure 4A shows the sEPSCs traces in spinal dorsal horn neurons from three groups of rats. Cumulative distributions shows that the curve of sEPSC interevent interval was significantly shifted rightward in paclitaxel-metformin (i.p.)-treated (MET) rats, compared with those from paclitaxel-treated (PAC) rats (Figure 4B). However, no change in the curve of sEPSC amplitude was observer between the two groups of rats (Figure 4D). Figures 4C, E show that intraperitoneal injection of metformin also significantly decreased the increased frequency of sEPSCs (1.18 ± 0.37 Hz vs. 1.89 ± 0.42 Hz, *P* < 0.01, *n* = 6 neurons from 6 rats), but did not affect the sEPSC amplitude in spinal dorsal horn neurons from paclitaxel-treated rats. The results indicated that systemic treatment with metformin also suppressed the increased frequency of sEPSCs in spinal dorsal horn neurons from paclitaxel-treated rats.

## 4. Discussion

This study showed that mechanical allodynia was induced by intraperitoneal injection of paclitaxel and reached its peak within the 7th day to the 15th day after paclitaxel administration. We demonstrated that consecutive intrathecal injection of metformin at day 8, 9, and 10 dose-dependently alleviated the established mechanical allodynia in paclitaxel treated rats. Consistently, spinal and systemic application of metformin could suppress the potentiated spinal synaptic transmission in paclitaxel-treated rats.

It has been well documented that enhanced neuronal activation in the spinal dorsal horn is involved in a variety of pathological pain (Woolf, 2011). These include neuropathic pain induced by sciatic nerve-injury, diabetes, paclitaxel or vincristine chemotherapy (Weng et al., 2003; Li et al., 2010; Nie and Weng, 2010; Yan et al., 2015). Our results showed the frequency rather than the amplitude of sEPSCs in superficial dorsal horn neurons was increased in spinal cord slice from paclitaxel-treated rat. Previous studies have shown that the nociceptive synaptic transmission is enhanced by intraperitoneal injection of paclitaxel, which contributes to chemotherapy-induced neuropathic pain (Zhu et al., 2015; Xie et al., 2016). We observed that the increased frequency of sEPSCs did not occur after consecutive intrathecal or systemic administration of metformin in paclitaxel-treated rats, suggesting metformin prevented the paclitaxel-induced potentiation of spinal synaptic transmission. The increased frequency of sEPSCs was also significantly suppressed after the spinal cord slices from paclitaxel-treated rats were pre-incubated with metformin for 1 h, indicating the short-term treatment with metformin has an acute effect on enhanced synaptic transmission. However, pretreatment with metformin decreases sEPSC amplitudes and has no effect sEPSC frequency in oxaliplatin-incubating spinal slices (Ling et al., 2017). The possible reason for this discrepancy is the different effects of oxaliplatin on spinal synaptic transmission. Oxaliplatin only increases sEPSC amplitudes in spinal cord slice without change in sEPSC frequency (Ling et al., 2017). The present and previous studies have shown that paclitaxel had a direct effect on the increasing frequency of EPSCs in the spinal cord (Li et al., 2015). It has been found that DRG neurons, including their terminals, are the targets of paclitaxel attack (Matsumoto et al., 2006; Scuteri et al., 2006; Hara et al., 2013; Li et al., 2021). These findings suggest that paclitaxel may interact with presynaptic terminals in the dorsal horn, but not directly with postsynaptic neurons and other spinal neurons.

The current results cannot reveal the mechanisms underlying inhibition of sEPSC frequency by metformin in paclitaxel-treated rat spinal cord slices. Recently, metformin has been reported to inhibit L-type voltage-dependent calcium channel (Wang et al., 2020). In spinal lamina, the L-type calcium channel is located at presynaptic terminal and involved in synaptic transmission (Kim et al., 2001; Qian et al., 2013). The L-type calcium channel blocker can reduce the frequency of sEPSCs in spinal lamina in peripheral nerve injury model (Alles et al., 2018). It needs to be further verified whether metformin prevented the enhanced synaptic transmission in paclitaxel-treated rat spinal cord slices through inhibition of L-type calcium channel.

The present study showed that intrathecal injection of metformin reversed the established mechanical allodynia produced by paclitaxel in rats, similar to its role in paclitaxel-induced and nerve injury-induced neuropathic pain (Melemedjian et al., 2011, 2013; Inyang et al., 2019). However, intraperitoneal injection of metformin could prevent development of mechanical allodynia induced by cisplatin and paclitaxel in mice only when the injection was started before the administration of these chemotherapeutics, and has no effect when metformin was injected after the cisplatin application (Mao-Ying et al., 2014). It is unclear whether the reason for this discrepancy is related to the route of administration and animal species. Most studies believe that the analgesic effect of metformin is mainly depends on the activation of AMP-activated protein kinase (AMPK), which can alleviate the nociceptive behavior of animals in a variety of pain models, such as those caused by nerve injury, surgical incision, diabetes neuropathy and chemotherapy (Melemedjian et al., 2011; Tillu et al., 2012; Mao-Ying et al., 2014; Ma et al., 2015; Maixner et al., 2015; Wang et al., 2018; Inyang et al., 2019). Decreased AMPK activity was found in the dorsal horn of the spinal cord in animals with partial sciatic nerve ligation. Spinal AMPK knockdown by siRNA results in behavioral hypersensitivity (Maixner et al., 2015). Especially, mice lacking AMPKα1 display increased glutamatergic synaptic activity in the spinal dorsal horn and mechanical allodynia (Maixner et al., 2016). These findings provide a clue that metformin may suppress the potentiated spinal synaptic transmission and relieve paclitaxel-induced neuropathic pain by activating spinal AMPK signaling, although it need to examine whether metformin could increase the expression of AMPK in the spinal dorsal horn of rats with the neuropathic pain.

## 5. Conclusions

In conclusions, metformin was able to depress the potentiated spinal synaptic transmission, which may contribute to alleviating the paclitaxel-induced neuropathic pain.

## Data availability statement

The original contributions presented in this study are included in the article/supplementary material, further inquiries can be directed to the corresponding author.

## Ethics statement

The animal study was reviewed and approved by the Animal Research Ethics Committee of Hubei University of Science and Technology (2016-03-005).

## Author contributions

W-PH designed the research. T-TL and C-YQ performed the experiments. T-TL participated in the data analysis. T-TL and W-PH wrote the manuscript. All authors contributed substantially to this research and reviewed this manuscript.
